# *TLR10* overexpression modulates immune response in A549 lung epithelial cells challenged with SARS-CoV-2 S and N proteins

**DOI:** 10.3389/fimmu.2024.1490478

**Published:** 2025-01-20

**Authors:** Špela Knez, Mojca Narat, Jernej Ogorevc

**Affiliations:** Department of Animal Science, Biotechnical Faculty, University of Ljubljana, Ljubljana, Slovenia

**Keywords:** SARS-CoV-2, COVID-19, Toll-like receptor 10, interferons, proinflammatory cytokines, innate immunity

## Abstract

Toll-like receptors (TLRs) play an important role in the recognition of viral particles and activation of the innate immune system, but their role in SARS-CoV-2 infection is still poorly characterized. In the present study, we investigated the role of Toll-like receptor 10 (TLR10) in modulating the immune response during SARS-CoV-2 infection. The results showed that overexpression of *TLR10* in A549 lung epithelial cells, immunostimulated with SARS-CoV-2 proteins S and N mainly downregulated proinflammatory cytokines and interferons and affected gene expression in the cocultured THP-1 monocytes. Our results suggest that TLR10 could mediate the extent of SARS-CoV-2 infection by downregulating the release of inflammatory cytokines and chemokines such as *CXCL10*, *IL6*, *IL8*, and *IFNβ*. Modulation of *TLR10* expression could have implications for the treatment of patients with severe COVID-19, in whom excessive inflammation leading to the development of acute respiratory distress syndrome (ARDS) is a key feature. However, further research is needed to fully understand the impact of modulating *TLR10* expression on the antiviral response and the overall balance of the immune response during SARS-CoV-2 infection.

## 1 Introduction

Severe acute respiratory syndrome coronavirus-2 (SARS-CoV-2) is a recently emerged virus that causes coronavirus disease 2019 (COVID-19). Since 2019, it has spread worldwide, affecting global health, social life and the world economy. SARS-CoV-2 is a β-coronavirus that belongs to the *Coronaviridae* family and is an enveloped positive-strand RNA (+ssRNA) virus. The virus consists of four main structural proteins, namely the spike glycoprotein (S), the small envelope glycoprotein (E), the membrane glycoprotein (M) and the nucleocapsid phosphoprotein (N), as well as several accessory proteins, such as the papain-like protease (P) (1).

Infection of the respiratory tract with SARS-CoV-2 leads to an extremely diverse clinical spectrum. It ranges from asymptomatic forms and mild symptoms such as fever, dry cough and shortness of breath to severe symptoms that require hospitalization. In the most severe cases of SARS-CoV-2 infection, acute overproduction and uncontrolled release of proinflammatory cytokines (hypercytokinemia, also known as “cytokine storm”) can trigger excessive systemic inflammation leading to acute respiratory distress syndrome (ARDS) and multiple organ failure (2). The severity of the disease has been associated with elevated levels of various proinflammatory cytokines and chemokines, such as interleukin (IL)1β, IL6, IL8, C-X-C motif chemokine ligand 10 (CXCL10), chemokine (C-C motif) ligand 20 (CCL20), tumor necrosis factor alpha (TNFα), and a delayed type I interferone (IFN) response (3–6).

Airway epithelial cells interface with the external environment and play a key role in recognizing airborne pathogens and activating the host immune system (7). Pattern recognition receptors (PRRs) such as Toll-like receptors (TLRs), which recognize pathogen-associated molecular patterns (PAMPs) and trigger an immune response, are important components of the innate immune system. Members of the TLR family are the best-studied PRRs that can also recognize structural proteins of SARS-CoV-2 (8). TLRs on endosomes recognize intracellular components of viruses; for example, TLR7 and TLR8 can recognize viral single-stranded RNA (9, 10), TLR3 recognizes transient dsRNA intermediates formed during replication of the virus (11), while TLRs on the cell membrane recognize viral proteins. The S protein is thought to be recognized by both TLR2 (12) and TLR4 (13), while protein E is recognized by TLR2 (14). It has been shown that not only the viral infection but also various individual SARS-CoV-2 proteins are immunogenic both *in vivo* and *in vitro* (15) and trigger the expression of proinflammatory cytokines in immune (14, 15) and epithelial cells (12).

TLR10 is the only poorly characterized member of the TLR family. It belongs to the TLR2 subfamily and can homodimerize in the presence of its respective ligands or act in paired combination with other members of the subfamily (TLR1, TLR2, TLR6) (16). To our knowledge, the role of TLR10 in the recognition of SARS-CoV-2 has not yet been investigated, but it is known that *TLR10* is expressed in lung epithelial cells (17). Our recent results confirmed that TLR10 is indeed present in A549 lung epithelial cells and showed that its overexpression by CRISPRa downregulates some of the proinflammatory mediators in cells immunostimulated with dsRNA, LPS or Pam3Cys (18). Studies also show that upregulation of *TLR10* is possible by adding the biologically active form of vitamin D, calcitriol (1,25-dihydroxycholecalciferol), to the cell growth medium (19). However, the effects of other vitamins (e.g. vitamin C and B vitamins) associated with the prevention and management of COVID-19 (20) on *TLR10* expression in cell cultures have not been determined. As several vitamins, especially D and C, have been associated with the prevention and treatment of diseases, including SARS-CoV-2 infections (e.g. 21, 22), we were interested in whether the addition of vitamins to the growth medium affects *TLR10* expression; if so, one could speculate that the immunomodulatory effects of vitamins could be explained, at least in part, by their effect on the regulation of TLR10 expression.

The aim of this study was to investigate the potential immunoregulatory effects of *TLR10* upregulation in the A549 cell line immunostimulated with SARS-CoV-2 S and N proteins. To our knowledge, this is one of the first studies to address the potential immunomodulatory effects of TLR10 in lung epithelial cells during immunostimulation with SARS-CoV-2 proteins in an *in vitro* system. Harnessing the immunomodulatory effects of TLR10 could have potential therapeutic applications for the treatment of patients with severe COVID-19 as well as for the management of other inflammatory diseases.

## 2 Materials and methods

### 2.1 Cell culture

The human lung epithelial cell line A549 was purchased from the American Type Culture Collection (ATCC). Cells were grown in DMEM (Sigma Aldrich) supplemented with 10% FBS (Sigma–Aldrich), 2 mM L-glutamine (Thermo Fisher Scientific), and 0.01% penicillin-streptavidin (Sigma–Aldrich) at 37°C and 5% CO_2_.

The human mononuclear cell line THP-1 (ATCC) was grown in RPMI 1640 medium (Sigma–Aldrich) supplemented with 10% FBS (Sigma–Aldrich) and 0.01% penicillin-streptavidin (Sigma–Aldrich) at 37°C and 5% CO_2_.

### 2.2 *TLR10* overexpression

Endogenous *TLR10* was overexpressed in A549 cells with CRISPR/dCas9 as previously described (18). Briefly, cells were transfected using Lipofectamine 3000 (Thermo Fisher Scientific) at approximately 70% confluence with the vectors pCMV-dCas9:NLS: VPR (23), encoding dCas9-VPR, and pGGAselect (N0309AAVIAL, New England BioLabs), encoding sgRNAs for *TLR10* overexpression, (A549-TLR10_OE_) or with an empty pGGAselect vector representing *TLR10* control expression (A549_C_). To avoid cell apoptosis, cells were washed with PBS four hours after transfection, and the medium was changed. All experiments were performed 48 hours after transfection. Localization of TLR10 in A549_C_ and A549-TLR10_OE_ was determined using immunostaining (see **Supplementary Section S4**).

In addition, ascorbic acid (A4544, Merck), folic acid (F8758, Merck), calciferol (D1530, Merck) and riboflavin (R9504, Merck) were added to the growth medium of non-transfected A549 cells at two different concentrations (50 or 200 µM) to determine their effects on *TLR10* expression after 24 and 48 hours of incubation.

### 2.3 Immunostimulation of A549 cells

Cells were immunostimulated with final concentrations of 500 ng/mL SARS-CoV-2 protein S (RP87668, Thermo Fisher Scientific) or SARS-CoV-2 protein N (RP87707, Thermo Fisher Scientific) and incubated for four and 24 hours (corresponding to “early” and “late” immune response, respectively) to simulate immune challenge of lung epithelial cells during SARS-CoV-2 infection. RNA was extracted from the cells and gene expression was quantified by real-time PCR. In addition, the growth media were collected for the quantification of cytokines.

### 2.4 Co-culture of stimulated A549-TLR10_OE_ cells and THP-1 cells

**Figure 1:**
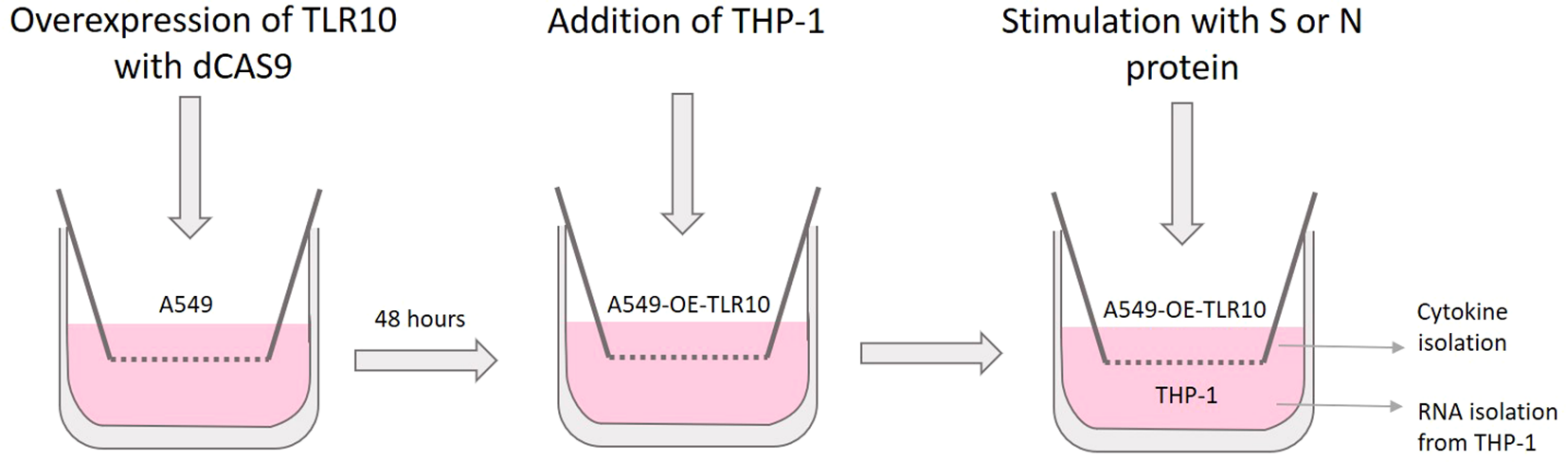
Schematic representation of the co-culture experiments involving A549 and THP-1 cells in a transwell system.

Cells were plated on 12 mm Transwell™ plates with 0.4 μm pore polyester membrane inserts (3460, Corning). One day before transfection, A549 cells were seeded at a concentration of 1×10^5^ cells/well in the upper chamber of the Transwell system (**Figure 1**). On the day of transfection, cells were transfected with pCMV-dCas9:NLS: VPR and pGGAselect as described above to obtain A549-TLR10_OE_ and A549_C_ cells. After four hours of incubation, the cells were washed with PBS and the medium was changed. After two days, THP-1 cells were seeded into the lower chamber at a concentration of 0.5×10^6^ cells/well. A549-TLR10_OE_ and A549_C_ cells were stimulated with SARS-CoV-2 S or N protein at a final concentration of 500 ng/mL for four or 24 hours, respectively. The cell culture medium was collected and RNA was isolated from THP-1 cells as described above.

### 2.5 RNA isolation and cDNA library preparation

Total RNA was isolated using the RNeasy Mini Kit (Qiagen) according to the manufacturer’s instructions. The extracted RNA was diluted to a final concentration of approximately 100 ng/µL RNA and treated with DNase I (Thermo Fisher Scientific). The treated RNA was reverse transcribed into single-stranded cDNA using a High Capacity cDNA Reverse Transcription Kit (Thermo Fisher Scientific) and stored at -20°C.

### 2.6 Gene expression analyses

#### 2.6.1 Digital PCR

Digital PCR (dPCR) was performed using a QIAcuity instrument (QIAGEN). Reactions were prepared using a QIAcuity Probe PCR Kit (QIAGEN) in a volume of 40 μL. Gene expression experiments were performed in duplicate. The expression of the target gene *TLR10* (Hs01935337_s1) and the reference gene *TOP1* (Hs00243257) was measured using TaqMan Gene Expression Assays (Thermo Fisher Scientific). Reactions using TaqMan probes at a final concentration of 0.8 µM were loaded onto a 26k nanoparticle dPCR nanoplate (QIAcuity) and performed as follows: 2 min, 95°C, 40× (15 s at 95°C, 15 s at 60°C). QIAcuity analysis software (QIAGEN) was used to analyze the results. Poisson distribution was used to calculate the number of copies of the target molecule per positive partition. To normalize the data, the copies/unit RNA of the target gene were multiplied by the normalization factor (geometric mean of TOP1), and the final data were displayed as normalized copies/unit RNA.

#### 2.6.2 Pathway-focused gene expression profiling with qPCR arrays

The cDNA was mixed with PowerUp™ SYBR^®^ Green Master Mix (Applied Biosystems) and aliquoted into the wells of a pre-made qPCR profiling array (RT^2^ Profiler PCR Array, GeneGlobe ID-PAHS-018Z, Qiagen) containing a panel of 84 genes associated with the TLR signaling pathway. Amplification results were normalized to the five housekeeping genes *ACTB*, *B2M*, *GAPDH*, *HPRT1*, and *RPLP0*. Fold changes in gene expression between immunostimulated A549_C_ and immunostimulated A549-TLR10_OE_ cells were calculated using the 2^−ΔΔCT^ method (24).

#### 2.6.3 Reverse transcription quantitative PCR

Further expression analyses were performed for individual genes identified as differentially expressed during qPCR array screening and/or consistently associated with the SARS-CoV-2 immune response in the literature. Primers were designed for the genes of interest using the online tool PrimerBlast (https://www.ncbi.nlm.nih.gov/tools/primer-blast/index.cgi (accessed May 27, 2023) (**Supplementary Table S1**). All the reactions were performed in triplicate. Thermal cycling conditions were as follows: 10 min at 95°C, 40 cycles at 95°C for 15 s and at 60°C for 1 min. Primer efficiencies were determined for all the primer pairs using a six-log cDNA dilution range. All the determined primer pair efficiencies were in the range of 100 ± 10% and the R^2^ ≥ 0.99. Relative differential expression between immunostimulated A549_C_ and immunostimulated A549-TLR10_OE_ cells was calculated using the 2^-ΔΔCt^ method (24). A gene was considered differentially expressed if it showed at least a two-fold change.

### 2.7 ELISA cytokine assays

The cell culture medium of A549 and THP-1 cells was removed four and 24 hours after incubation with SARS-CoV-2 proteins S and N, respectively, and centrifuged at 1000 × g for 10 minutes at 4°C. The concentrations of IL8 (assay range: 15.6-1,000 pg/mL, BMS204-3INST, Thermo Fisher Scientific), IL1β (assay range: 7.8-500 pg/mL, BMS224INST, Thermo Fisher Scientific), CXCL10 (assay range: 3.1-200 pg/mL, BMS284INST, Thermo Fisher Scientific), IFNβ (assay range: 50-4000 pg/mL, 414101, Thermo Fisher Scientific), IL10 (assay range: 50-4000 pg/mL, BMS215INST, Thermo Fisher Scientific), CCL20 (assay range: 0.8-600 pg/mL, EHCCL20, Thermo Fisher Scientific) and TNFα (assay range: 7.8-500 pg/mL, BMS223INST, Thermo Fisher Scientific) were measured according to the manufacturer’s instructions.

### 2.8 Western blot

Approximately 5×10^5^ A549 cells were seeded in 6-well plates and transfected with plasmids to overexpress *TLR10* as previously described. After two days, cells were lysed in ice-cold RIPA lysis buffer supplemented with protease and phosphatase inhibitor cocktails (4906845001, Roche). Protein concentration was determined using the Pierce BCA Protein Assay Kit (# 23227, Thermo Fisher Scientific) according to the manufacturer’s instructions. Approximately 15 μg of the cell lysate was transferred to a precast NuPAGE 4-12% gel (NP0321BOX, Thermo Fisher Scientific), electrophoresed at 180 V for two hours and immunoblotted onto a PVDF membrane. Membranes were stained with primary antibodies against TLR10 (1:1000) (PRS3275, Sigma–Aldrich) and β-actin (1:5000) (MA1-140, Thermo Fisher Scientific). Secondary HRP-conjugated anti-rabbit antibody (1:5000) (31460, Thermo Fisher Scientific) and HRP-conjugated anti-mouse antibody (1:5000) (31430, Thermo Fisher Scientific) were used for visualization, and the HRP signal recorded after two hours of incubation with TrueBlue peroxidase substrate (5510-0030, LGS Diagnostics).

### 2.9 Statistical analysis

Data were analyzed using Prism 8 version 10.2.2 software (GraphPad). Statistical significance was determined using a two-tailed, unpaired Student’s t-test (p ≤ 0.05 was considered statistically significant).

## 3 Results

Overexpression of endogenous *TLR10* in immunostimulated A549 cells significantly altered the expression of several cytokines and genes associated with TLR signaling. Furthermore, overexpression of *TLR10* in A549 cells co-cultured with THP-1 cells altered the expression of genes in THP-1 cells.

### 3.1 TLR10 upregulation

**Figure 2:**
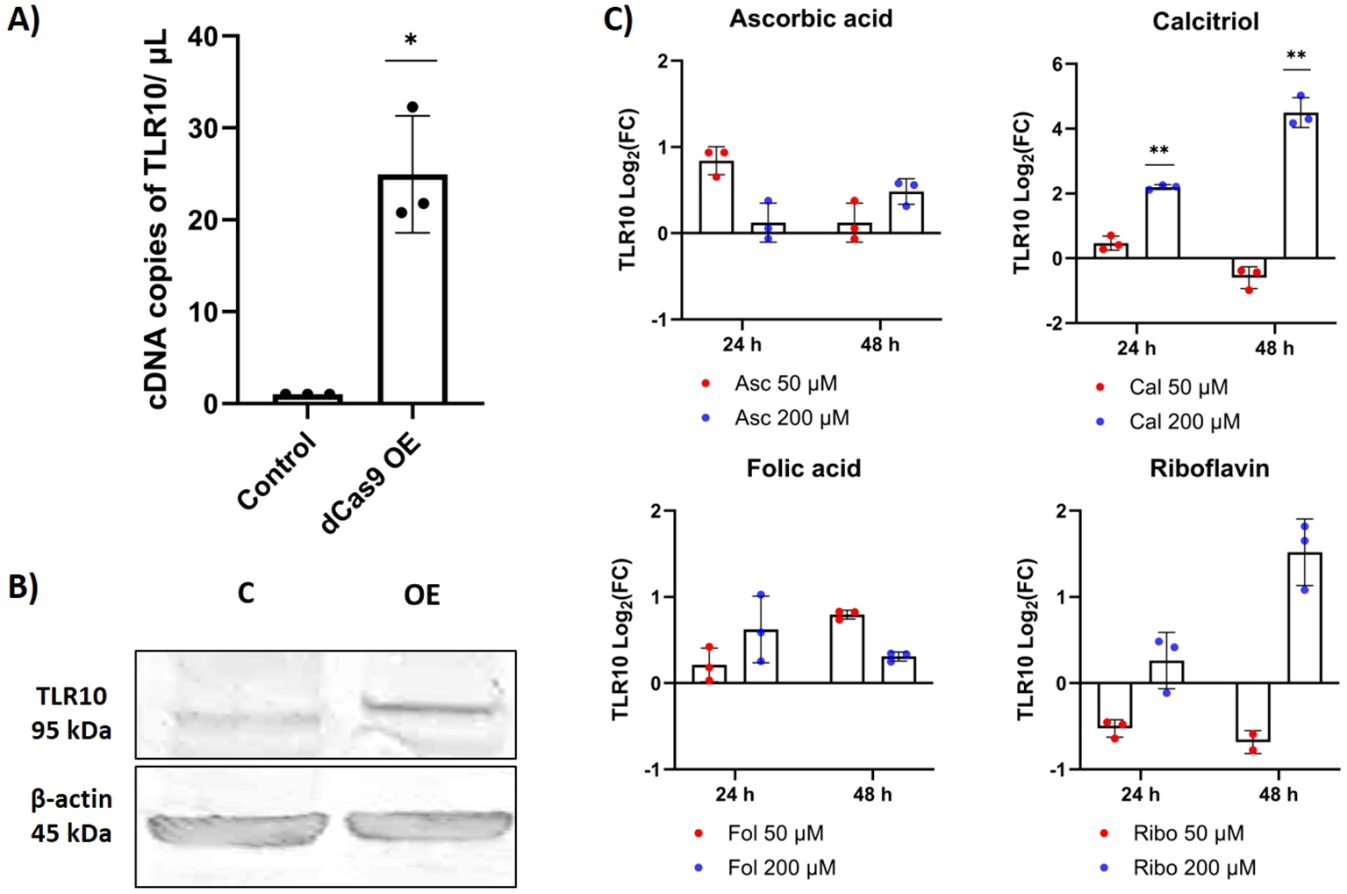
Differential expression of TLR10. **(A)** Differential expression of *TLR10* induced by CRISPR/dCas9, evaluated by dPCR. **(B)** Western blot showing TLR10 protein 48 hours after transfection. **(C)** Differential expression of *TLR10* after addition of different vitamins to the cell growth medium, evaluated by qPCR. Data are presented as mean with SEM of three independent experiments; *p ≤ 0.05, **p ≤ 0.01 by unpaired Student’s t-test.

Using the CRISPR/dCas9 system, we were able to increase the expression of *TLR10* by approximately 24-fold (**Figure 2A**). The induction appears to translate to the protein level, which is visible in the Western blot (**Figure 2B**; **Supplementary Figure S1**). After overexpression, TLR10 was mainly localized at the cell membrane, but to a lesser extent also in the cytoplasm (possibly in endosomes) (**Supplementary Figure S2**). Supplementation of the growth medium with calcitriol (active form of vitamin D) significantly affected *TLR10* expression, while supplementation with other vitamins (ascorbic acid – vitamin C, folic acid – vitamin B9, and riboflavin – vitamin B2) had no statistically significant effect on the expression of *TLR10* at any of the concentrations used. The addition of the biologically active form of vitamin D to the growth medium at a high concentration (200 µM) increased the expression of *TLR10* approximately four-fold after 24 hours of incubation and approximately 16-fold after 48 hours of incubation (**Figure 2C**).

### 3.2 Differential expression of immune-associated genes in A549 and THP-1 cells challenged with SARS-CoV-2 proteins S and N

**Figure 3:**
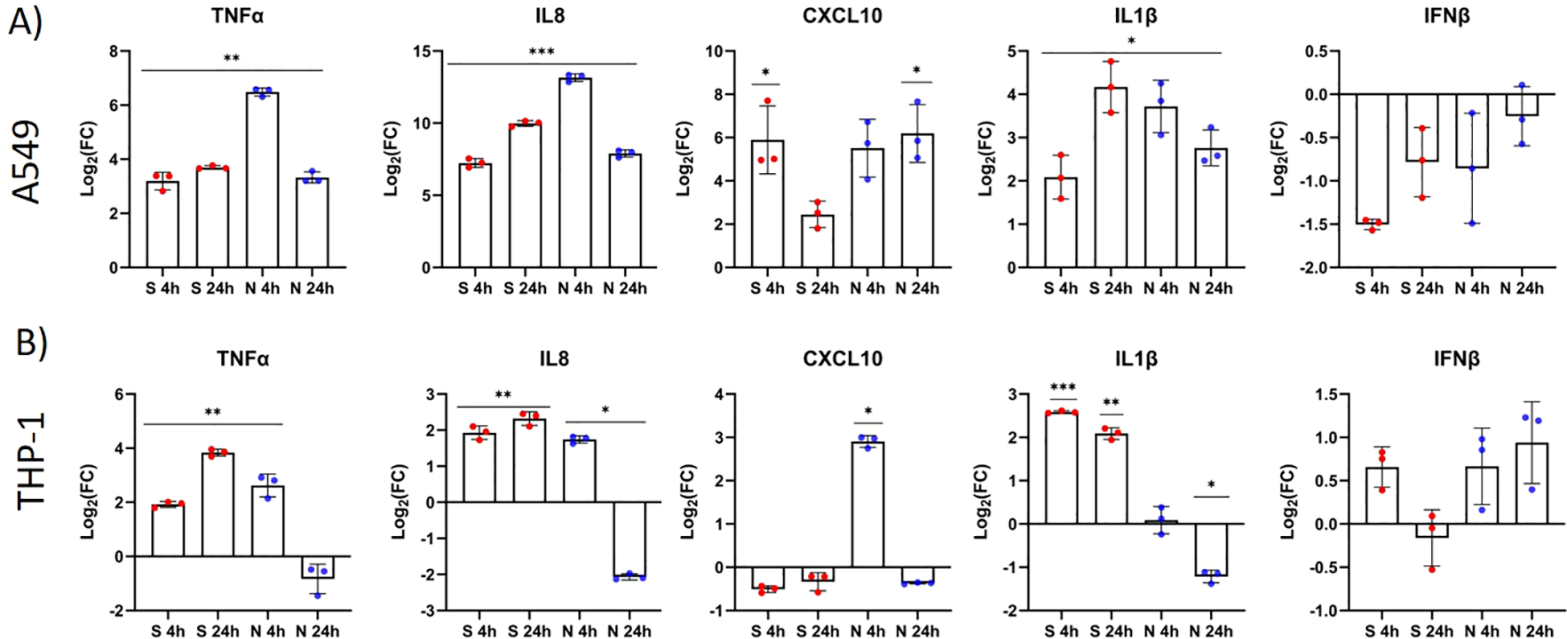
The S and N proteins of SARS-CoV-2 induce the differential expression of inflammatory markers (cytokines) in A549 and THP-1 cells. A549 cells **(A)** and THP-1 cells **(B)** were stimulated with the SARS-CoV-2 protein S (red) or N (blue) (both 500 ng/mL) for four and 24 hours. Gene expression of *TNFα*, *IL8*, *CXCL10*, *IL1β* and *IFNβ* was measured by RT–qPCR and compared between immunostimulated and untreated cells. Data are presented as mean with SEM of three independent experiments; *p < 0.05, **p < 0.01, ***p < 0.001 according to unpaired Student’s t tests.

Both S and N protein induced the expression of the proinflammatory cytokines *TNFα* and *IL1β* and the chemokines *CXCL10* and *IL8*, while *IFNβ* was downregulated in A549 cells (p > 0.05). In general, N protein induced the relative expression of inflammatory mediators in A549 cells earlier than S protein as shown by the expression data for four- and 24-hour time points after immunostimulation (**Figure 3**). The highest differential expression in immunostimulated A549 cells was observed for *IL8* and *CXCL10*, for both virulence factors, and for *TNFα* in the case of N protein challenge after four hours (**Figure 3A**). The induction of inflammatory mediators in THP-1 cells was weaker than in A549 cells. The S protein increased the expression of *TNFα*, *IL8* and *IL1β* at both time points after stimulation, while N protein induced the expression of these markers only in the “early” phase of immune stimulation (after four hours) (**Figure 3B**). No statistically significant difference in *IFNβ* expression was observed after immunostimulation with the S or N protein.

### 3.3 Immunomodulatory effects of TLR10 overexpression in A549 cells immunostimulated with SARS-CoV-2 S protein

**Figure 4:**
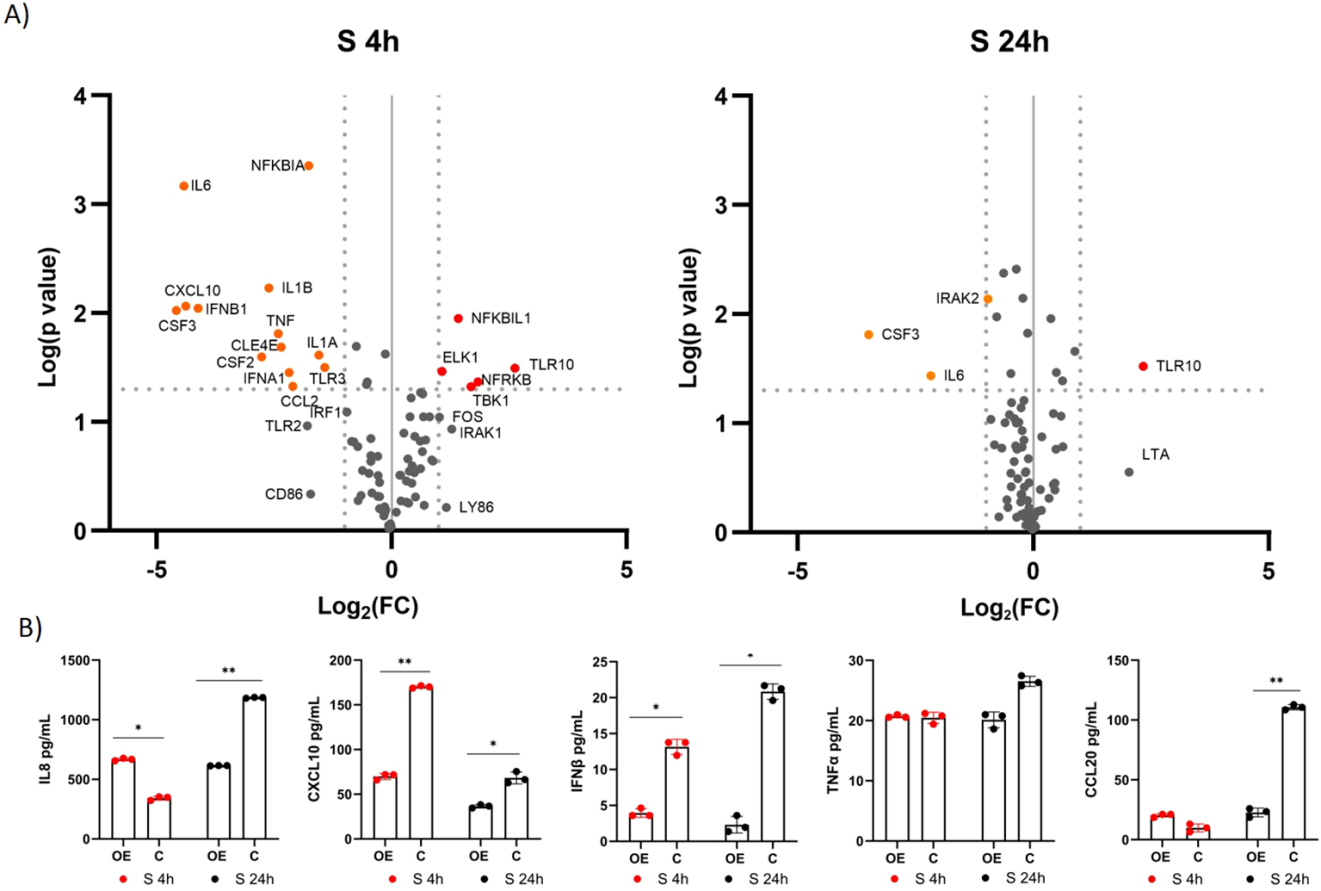
Differential expression of immune-associated genes in A549-TLR10_OE_ cells challenged with the S protein. **(A)** a volcano plot of differential expression between immunostimulated A549-TLR10_OE_ and immunostimulated A549_C_ cells challenged for four and 24 hours as determined by qPCR arrays. Log2-fold changes are plotted against the p-values of the t-test. Significantly downregulated genes are marked as orange dots, while significantly upregulated genes are marked as red dots. Fold-change (≥ 2-fold, vertical lines) and statistically significant difference (p ≤ 0.05, horizontal line) were used as threshold for differential expression. **(B)** Cytokine secretion was measured by ELISA. Data are presented as mean with SEM of three independent experiments; *p ≤ 0.05, **p ≤ 0.01 by unpaired Student’s t-test. OE, overexpressed; C, control.

Using a qPCR array focusing on genes associated with the TLR signaling pathway, we investigated the differential expression of 84 genes involved in this pathway. We compared the expression of the genes in immunostimulated cells overexpressing *TLR10* (A549-TLR10_OE_) with immunostimulated cells with native *TLR10* expression (A549_C_) (**Supplementary Table S2**; **Figure 4A**). Gene expression analysis revealed five upregulated (*NFRKβ, TBK1, TLR10, NFKβIL1, IRAK1*) and 13 downregulated (*CSF3, IL6, CXCL10, IFNβ1, CSF2, IL1β, TNFα, CLE4E, IFNα, CCL2, NFKβIA, IL1A, TLR3*) genes four hours after stimulation with the S protein (≥ 2-fold change and a p-value ≤ 0.05 was used as a threshold for differential expression). Prominent immune mediators such as *IL6*, *CXCL10*, *IL1β*, *TNFα*, *IFNβ* and the colony-stimulating factors *CSF2* and *CSF*3 were downregulated, while genes encoding adaptor proteins, kinases and transcription factors such as *IRAK1*, *TBK1*, *FOS* and *ELK* were upregulated in response to *TLR10* overexpression. Twenty-four hours after stimulation, the effect of *TLR10* overexpression diminished, as only three genes were downregulated (*IL6*, *CSF3* and *IRAK2*).

To further investigate the effects of *TLR10* overexpression, protein concentrations of known inflammatory markers, namely, IL8, CXCL10, IFNβ, TNFα, CCL20, and IL1β, were measured by ELISA (**Figure 4B**). The concentrations of the cytokines in the medium were not entirely consistent with the qPCR results, e.g. TNFα levels were not significantly different between the treatment and the control, while IL8 levels were elevated in the sample overexpressing *TLR10* at four hours after immunostimulation. Additionally, we were unable to detect IL1β in the growth medium, while the measured IFNβ levels were below the range value of the assay (50 pg/mL).

### 3.4 Immunomodulatory effects of TLR10 overexpression in A549 cells immunostimulated with SARS-CoV-2 N protein

**Figure 5:**
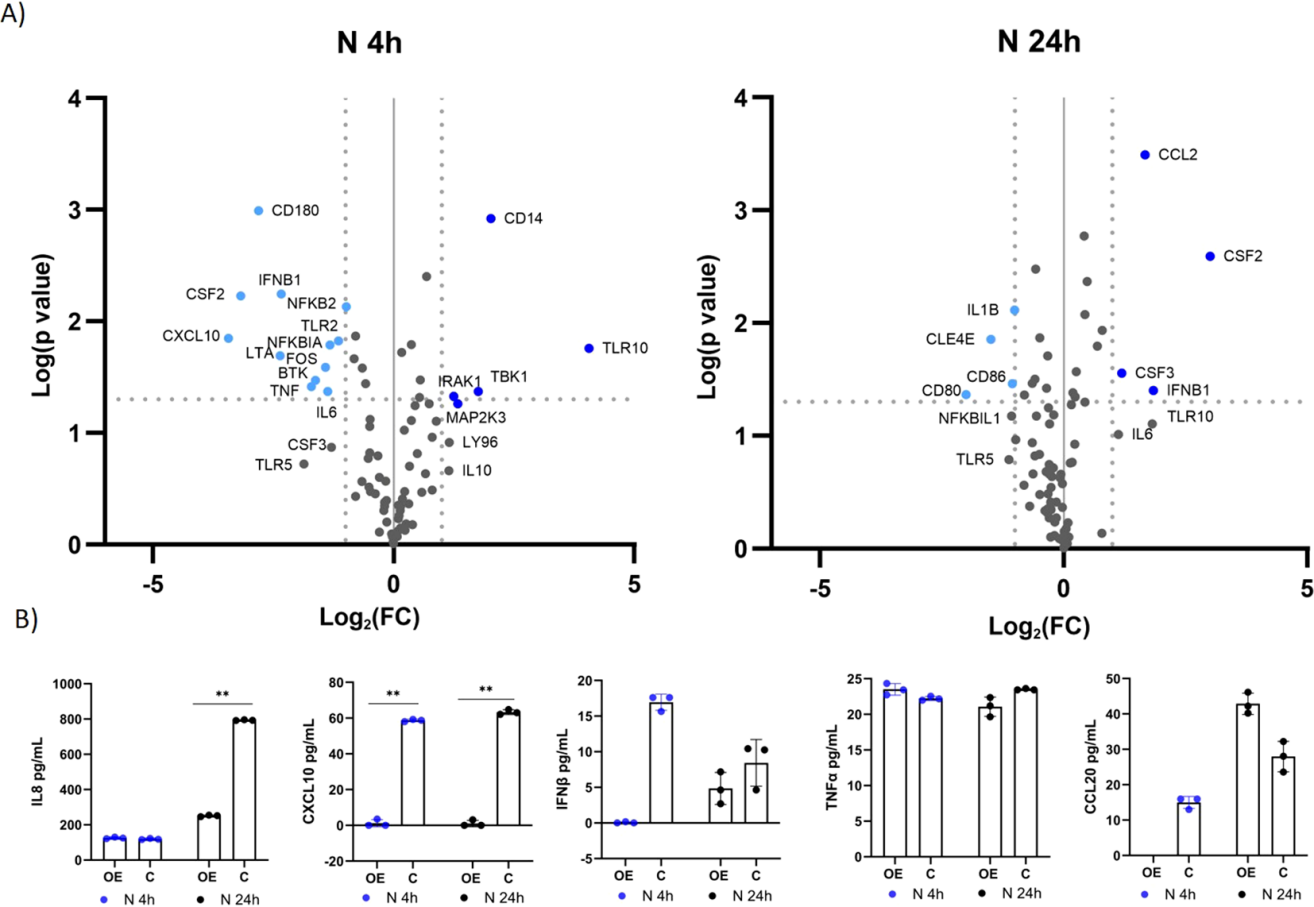
Differential expression of immune-associated genes in A549-TLR10_OE_ challenged with the N protein. **(A)** a volcano plot of differential expression between immunostimulated A549-TLR10_OE_ and immunostimulated A549_C_ cells challenged for four and 24 hours as determined by qPCR arrays. Log_2-_fold changes are plotted against the p-values of the t-test. Significantly downregulated genes are marked as light blue dots, while significantly upregulated genes are marked as dark blue dots. Fold-change (≥ 2-fold, vertical lines) and statistically significant difference (p ≤ 0.05, horizontal line) were used as threshold for differential expression. **(B)** Cytokine secretion was measured by ELISA. Data are presented as mean with SEM of three independent experiments; **p ≤ 0.01 by unpaired Student’s t-test. OE, overexpressed; C, control.

A similar response to immunostimulation with S protein was observed after immunostimulation with N protein (**Supplementary Table S2**; **Figure 5A**). After four hours, 12 genes were downregulated (*CD180, CXCL10, CSF2, CD180, LTA, IFNβ1, TNFα, BTK, FOS, IL6, NFKBIA, TLR2*), and five were upregulated (*CD14, TLR10, TBK1, IRAK1, MAP2K3*) in response to *TLR10* overexpression. After 24 hours, the effect of *TLR10* overexpression diminished, as only four genes were downregulated (*IL1β, CLE4E, CD86*, and *CD80*) and four were upregulated (*CSF2, CSF3*, and *IFNβ1*). Cytokine secretion was measured by ELISA for IL8, CXCL10, IFNβ, and TNFα, CCL20, and IL1β (**Figure 5B**). The concentrations of cytokines in the medium were not entirely consistent with the qPCR results, for example concentrations of TNFα were not significantly different between treatment and control, while CCL20 levels were elevated in the sample overexpressing *TLR10* 24 hours after immunostimulation. We were unable to detect IL1β in the growth medium, while the measured IFNβ levels were below the range value of the assay (50 pg/mL).

### 3.5 TLR10 overexpression in immunostimulated A549 cells modulates gene expression of THP-1 monocytes in co-culture

**Figure 6:**
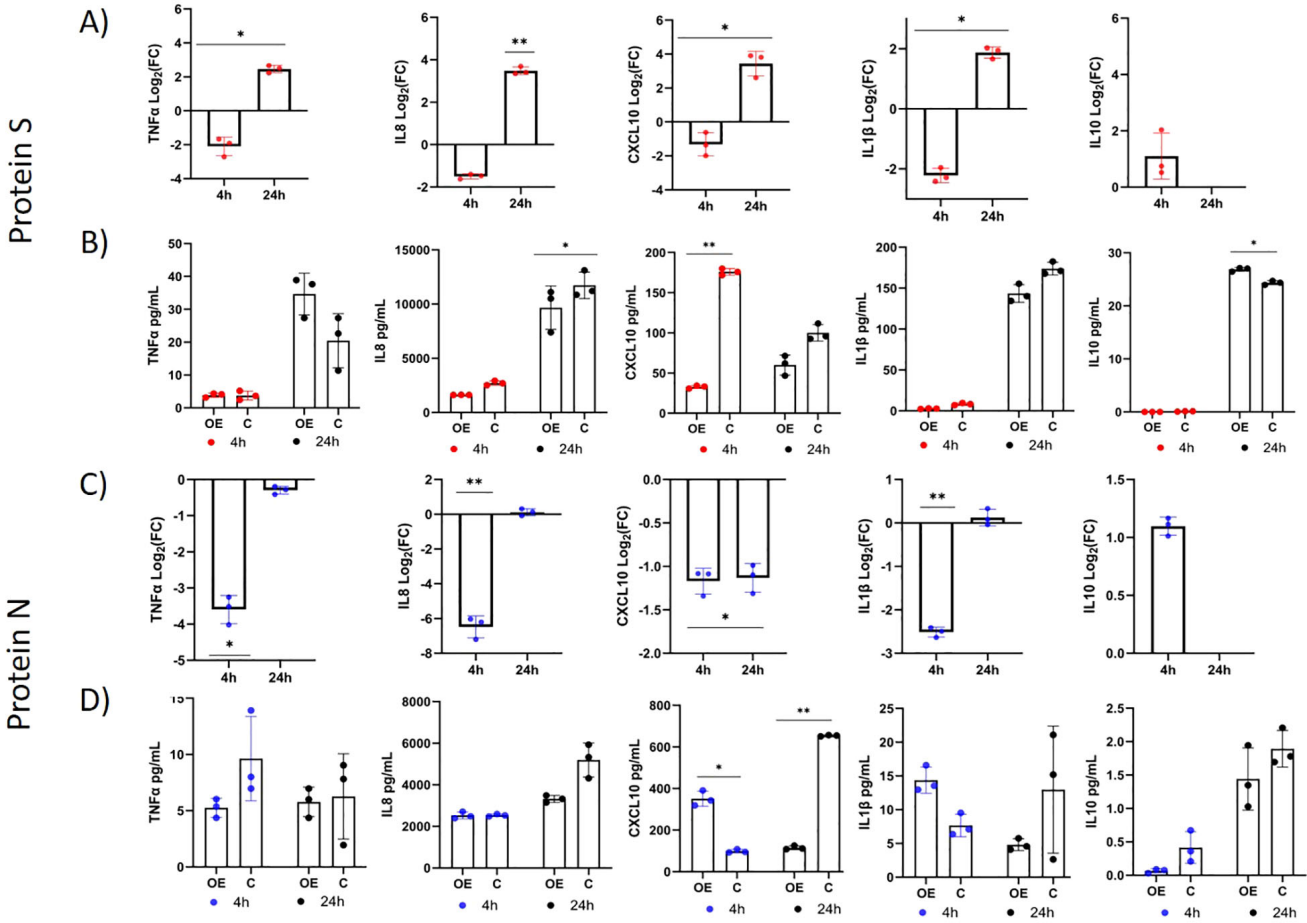
Differential expression of immune-associated genes in THP-1 cells co-cultured with immunostimulated A549-TLR10_OE_ or immunostimulated A549_C_ cells (A549 cells were challenged with the N or S protein for four and 24 h). **(A, C)** The differential expression of *TNFα*, *IL8*, *CXCL10*, *IL10* and *IL1β* in THP-1 cells between coculture with immunostimulated A549-TLR10_OE_ and immunostimulated A549_C_ cells. **(B, D)** The protein concentrations of TNFα, CXCL10, IL10, IL1β, and IL8 in the co-culture medium measured by ELISA. Data are presented as mean with SEM of three independent; *p ≤ 0.05, **p ≤ 0.01 by unpaired Student’s t-test. OE, overexpressed; C, control.

In the co-culture experiment, we investigated whether overexpression of *TLR10* in immunostimulated A549 cells affects gene expression in the monocytic human leukemia cell line THP-1. The results showed that overexpression of *TLR10* in immunostimulated A549-TLR10_OE_ altered expression of genes in co-cultured THP-1, by downregulating expression of various proinflammatory cytokines (*TNFα*, *IL8*, *CXCL10*, and *IL1β*) four and 24 hours after immunostimulation with N protein and after four hours in the case of S protein, while their expression was upregulated 24 hours after immunostimulation with S protein (**Figures 6A, C**). In response to *TLR10* overexpression in immunostimulated A549 cells, granulocyte-macrophage colony-stimulating factors 2 (*CSF2*) and *CSF3*, which activate monocytes to produce inflammatory cytokines, were downregulated in A549-TLR10_OE_ (**Figures 4A**, **5A**) and in A549-TLR10_OE_ co-cultured with THP-1.

The overexpression of *TLR10* affected the concentrations of cytokines in the co-culture medium (**Figures 6B, D**). In the case of immunostimulation with S protein (**Figure 6B**), the concentrations of IL8 (concentrations in some samples exceeded recommended assay range value of 1000 pg/mL), IL1β and CXCL10 were lower in the case of *TLR10* overexpression at both time- points after immunostimulation, while the concentrations of IL10 and TNFα were higher 24 hours after immunostimulation and undetectable four hours after stimulation. In the case of four-hour immunostimulation with N protein, the concentrations of the cytokines CXCL10 and IL1β were higher in the A549-TLR10_OE_ co-culture medium than in the A549_C_ co-culture medium. However, 24 hours after immunostimulation the levels of all the cytokines were lower in the A549-TLR10_OE_ co-culture medium (significantly in case of CXCL10). The concentrations of TNFα and IL10 were lower in the A549-TLR10_OE_ co-culture medium than in the A549_C_ co-culture medium at both time points after immunostimulation with N protein (p > 0.05), but the measured levels of IL10 and TNFα (in some of the samples) were below the recommended range values for the assays (7.8 pg/mL for TNFα and 50 pg/mL for IL10).

## 4 Discussion

Although studies have shown that some TLRs recognize SARS-CoV-2 and trigger an inflammatory response, the role of TLR10 in SARS-CoV-2 infection has not yet been investigated. TLR10 is considered an orphan receptor without a clear function, a specific ligand or a specific mode of action (16). Since mice lack a functional TLR10 receptor (25), studies on TLR10 have mainly been conducted *in vitro* and have yielded contradictory results. Depending on cell type, ligand, expression type and dimerization (homo or heterodimers), TLR10 has been associated with either a proinflammatory (26, 27) or an anti-inflammatory function (28, 29). TLR10 forms heterodimers with the TLR2 subfamily; TLR10-TLR1/TLR6 dimers have been shown to have proinflammatory effects (30), while TLR10-TLR2 dimers have been associated with anti-inflammatory effects (25). Recent studies have shown that TLR10 is involved in triggering the innate immune response against viruses by recognizing dsRNA (31) as well as viral proteins such as HIV-1 (32) and influenza virus proteins (33). A study by Zheng et al. (14) showed that TLR2 and MyD88 are required for the inflammatory response induced by β-coronaviruses (14). Therefore, we hypothesized that TLR10 may be involved in immune signaling in response to certain SARS-CoV-2 structural proteins, possibly through heterodimerization with TLR2.

To investigate the potential role of TLR10 in the inflammatory response induced by SARS-CoV-2 proteins in lung epithelial cells, we overexpressed endogenous *TLR10* in A549 cells and immunostimulated the cells with SARS-CoV-2 S or N protein. In addition to CRISPR-induced activation (CRISPRa) we were able to successfully induce expression of endogenous *TLR10* by supplementing the growth medium of challenged A549 cells with an active form of vitamin D. It is known that the active form of vitamin D interacts with the nuclear vitamin D receptor (VDR). VDR then forms a heterodimer with retinoid-X-receptor alpha (RXR-α) and the complex can bind to the vitamin D response elements located in the promoter regions of the target genes. Three potential VDR binding sites have been identified within the proximal promoter of *TLR10*, located within 250 base pairs upstream of the transcription start site (34). The interaction of vitamin D with TLR10 could potentially influence the body’s defense mechanisms against viral infections, including SARS-CoV-2. Further research is needed to fully understand the effects of this signaling pathway in the context of COVID-19 and other viral infections.

Studies on serum cytokines in patients with COVID-19 have shown that the concentrations of many cytokines and chemokines, such as IL6, TNFα, CSFs, monocyte chemoattractant proteins (MCPs) and CXCL10 (5, 35), are elevated in response to SARS-CoV-2 infection and correlate positively with the severity of the disease (36, 37). Therefore, downregulation of the aforementioned markers could alleviate the symptoms of COVID-19 and prevent hyperinflammation. First, we investigated whether the S or N protein triggers inflammatory signaling pathways and cytokine production in A549 cells. We found that the presence of S or N protein in the medium caused differential expression of inflammatory genes. Next, we investigated whether the expression level of *TLR10* affects the expression of inflammatory mediators in immunostimulated A549 cells. We found that overexpression of *TLR10* downregulates the expression of several proinflammatory cytokines. The genes that were significantly (p < 0.05) downregulated in both cases (after stimulation with the S or N protein) at least one time-point after immunostimulation (four or 24 hours after immunostimulation) were *IL6*, *CXCL10*, *TNFα*, *IFNβ*, *CSF2*, and *NFKBIA*, while the only upregulated gene in both cases was *TBK1*.

The vast majority of inflammatory cells that infiltrate the lungs after infection are monocytes and macrophages (38, 39). Angiotensin converting enzyme 2 (ACE2), through which SARS CoV-2 enters target cells, is only weakly expressed in monocytes (40), and the signal for their activation during SARS-CoV-2 infections is thought to originate from paracrine signaling of infected tissue. Several known molecules involved in monocyte migration are TNFα, CCL2, IL1β, IL8, and CSFs (41, 42). The co-culture experiments with immunostimulated A549 and monocytic THP-1 cells were performed to investigate the effects of *TLR10* overexpression in the immunostimulated epithelial A549 cell line on TLR pathway-associated gene expression of immune cells (THP-1) in a co-culture cell model. Cell co-cultures contribute to a better understanding of complex biological processes, as a variety of signaling pathways and mechanisms require direct interactions between cells, including paracrine signaling through cytokines, growth factors, and transcriptional regulators that are activated or repressed in response to pathogens (43). Our results suggest that overexpression of *TLR10* in A549 epithelial cells influences cytokine and chemokine expression in co-cultured THP-1 cells. The response of THP-1 cells to *TLR10* overexpression in co-cultured A549 cells immunostimulated with S or N protein (**Figure 6**) was downregulation of several proinflammatory cytokines, consistent with previous reports (12). However, when the co-cultures were immunostimulated with S protein, we found that some of the cytokines (*IL8*, *CXCL10*, *IFNβ*, *TNFα*) in the THP-1 cells were initially downregulated in the “early” phase (4 hours) after immunostimulation and upregulated in the “late” phase (24 hours), but the concentrations of all measured cytokines (except of TNFα) were lower in A549-TLR_OE_ co-cultures than in A549_C_ co-cultures 24 hours after immunostimulation (**Figure 6B**). Furthermore, S protein immunostimulation of A549-TLR10_OE_ cells co-cultured with THP-1 led to a significant upregulation of the anti-inflammatory cytokine *IL10* in THP-1 cells. When N protein immunostimulated A549-TLR10_OE_ and THP-1 cells were co-cultured, the expression of inflammatory genes such as *IL8*, *CXCL10*, and *IL1β* was downregulated in THP-1 cells after 4 hours compared to A549_C_ co-cultures (**Figure 6C**), but the cytokine concentrations in the medium were not downregulated until 24 hours. Such reduced correlations between mRNA expression and protein levels could be explained by transcriptional delays – the time when transcription starts and when protein is formed (44, 45), which is why transcriptional regulation may not be immediately visible at the protein level.

In the present study, we demonstrated that TLR10 is able to modulate the immune response to SARS-CoV-2 proteins S and N in A549 and THP-1 cells. In general, we observed a downregulation of inflammatory cytokines when *TLR10* was overexpressed in immunostimulated A549 cells. However, some contradictory results (e.g. upregulation of some proinflammatory cytokines in immunostimulated A549-TLR10_OE_ cells compared to immunostimulated A549_C_ cells) and discrepancies between the transcriptional regulation of some genes and their protein levels were observed, especially in the »early« phase after immunostimulation, which may indicate that the mechanisms of TLR10 functions are complex and depend on virulence factor, incubation time, cell type, and TLR10 concentration. Furthermore, such discrepancies could be caused by the kinetics of CRISPRa regulation, as overexpression is not stable but decreases over time, as well as by possible epigenetic effects acting on RNA-mediated processes and preventing or delaying translation. In addition, we were unable to detect IL1β in A549 medium by ELISA or to optimize the dilutions of some of the samples so that their levels would fall within the recommended assay range (e.g. the measured IL10 concentrations in co-culture medium was below the recommended assay range value).

Our results suggest that TLR10 may mediate the extent of inflammation induced by SARS-CoV-2 by downregulating the release of inflammatory cytokines and chemokines such as CXCL10, IL6, IL8, and IFNβ. Whether the immunosuppressive effect of TLR10 on epithelial cells is desirable in patients with severe COVID-19 needs to be investigated in further experiments, as overexpression of *TLR10* also leads to downregulation of *IFNβ*, a key component of the antiviral response that is already suppressed by the elusive mechanism of SARS-CoV-2 (46). Delayed type-I interferon signaling promotes the accumulation of proinflammatory cytokines and chemokines, which may worsen the prognosis of patients. However, we believe that the immunomodulatory effects of TLR10 may represent an opportunity for intervention approaches to rebalance the excessive immune response at a later stage of infection, when an excess of inflammatory cytokines can become harmful to the tissue (cytokine storm).

Further studies are needed to determine the TLR10 ligands, to explain the mode of action by which it exerts immunomodulatory functions, and to determine the expression kinetics of target genes when CRISPRa is used. The lung is an organ suitable for drug delivery in aerosolized form by inhalation. Recent advances in inhalation strategies (47) may enable the development of inhalable liquid or powder CRISPR-based formulations for therapies and, in perspective, lead to the development of novel treatment strategies that utilize the immunomodulatory functions of TLR10.

## Data Availability

The datasets presented in this study can be found in online repositories. The names of the repository/repositories and accession number(s) can be found in the article/**Supplementary Material**.
