# Supplementary material for: TLR10 overexpression modulates immune response in A549 lung epithelial cells challenged with SARS-CoV-2 S and N proteins
Source: Front Immunol. 2025 Jan 20;15:1490478. doi: 10.3389/fimmu.2024.1490478 (PMC11788150; doi:10.3389/fimmu.2024.1490478)
Supplement: Supplementary file 1 [file DataSheet1.docx]

Supplementary material: TLR10 overexpression modulates immune response of A549 lung epithelial cells challenged with SARS-CoV-2 S or N protein

1.Target region specific primers

Supplementary table 1: Target region specific primers

| Gene | Forward | Reverse |
| --- | --- | --- |
| TLR10 | AGCCAACGACACTGGATT | ATGTTGCCAGCTTCCTCACA |
| TNFa | GGCGTGGAGCTGAGAGATAAC | GGTGTGGGTGAGGAGCACAT |
| IL8 | GAACTGAGAGTGATTGAGAGTGGA | CTCTTCAAAAACTTCTCCACAACC |
| CXCL10 | CCTGCAAGCCAATTTTGTCCA | TGTGTGGTCCATCCTTGGAA |
| IL1B | ATGATGGCTTATTACAGTGGCAA | GTCGGAGATTCGTAGCTGGA |
| IFNB | ATGACCAACAAGTGTCTCCT | CTGTCCTTGAGGCAGTATTC |
| IL10 | GACTTTAAGGGTTACCTGGGTTG | TCACATGCGCCTTGATGTCTG |
| TOP1 | CGCGCTCGTCCCTCC | AAATCCGCTTCGATCTGGGA |
| βACTIN | GGGACCTGACTGACTACCTC | AGCTTCTCCTTAATGTCACGC |
| GAPDH | GTCAGTGGTGGACCTGACCT | AGGGGTCTACATGGCAACTG |

2. Western blot

The cells were lysed with RIPA buffer, supplemented with the protease inhibitor cocktail (Sigma-Aldrich). Protein concentration of cell lysates were determined using Pierce BCA protein assay kit (Thermo Fischer) according to manufacturer’s instructions. 15 μg of protein lysates were loaded on an 8% SDS-PAGE gel (Thermo Fisher Scientific) and separated for two hours, and later transferred to a nitrocellulose membrane (GE Healthcare, Mississauga, CA). The membrane was blocked with 5% BSA in TBST for one hour to prevent nonspecific binding and incubated overnight with primary antibodies against TLR10 (1:1000) (Sigma-Aldrich) and β-actin (1:2000) (Thermo Fisher Scientific) at 4°C. For visualization, anti-rabbit-HRP conjugated antibodies (1:5000) (Thermo Fisher Scientific) were used and the results assessed two hours after incubation.


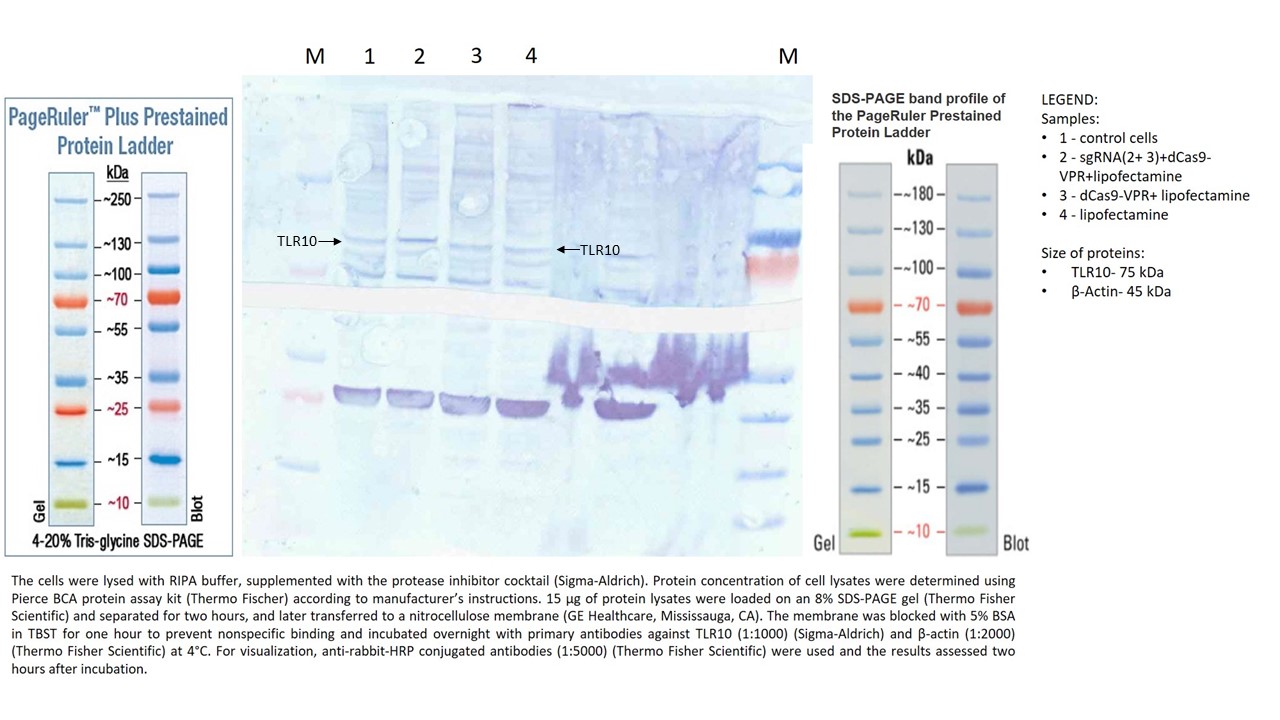


Supplementary figure 1: Original photo of western blot.

3. Pathway-focused gene expression profiling with qPCR arrays

Supplementary table 2: Expression analysis of genes related to the TLR biological pathway

| UniGene | GenBank | Symbol | Log_2_(TLR10_OE)_ | Log_2_(TLR10_OE_) | Log_2_(TLR10_OE_) | Log_2_(TLR10_OE_) | |
| --- | --- | --- | --- | --- | --- | --- | --- |
|  |  |  | **S, 4h** | **S, 24h** | **N, 4h** | **N, 24h** | |
| Hs.159494 | NM_000061 | BTK | 0.927 | 1.006 | -1.973 | -0.535 |  |
| Hs.599762 | NM_001228 | CASP8 | -0.529 | -0.145 | -0.719 | -0.146 |  |
| Hs.303649 | NM_002982 | CCL2 | -1.964 | -0.862 | -0.574 | 1.640 |  |
| Hs.163867 | NM_000591 | CD14 | 0.591 | 0.319 | 2.012 | 0.013 |  |
| Hs.87205 | NM_005582 | CD180 | -0.784 | NA | -2.928 | 1.012 |  |
| Hs.838 | NM_005191 | CD80 | 0.399 | 0.355 | 0.951 | -2.434 |  |
| Hs.171182 | NM_006889 | CD86 | -1.861 | -0.774 | NA | -0.883 |  |
| Hs.198998 | NM_001278 | CHUK | 0.558 | -0.071 | 0.202 | 0.049 |  |
| Hs.236516 | NM_014358 | CLEC4E | -2.458 | -0.160 | 0.012 | -1.505 |  |
| Hs.1349 | NM_000758 | CSF2 | -2.573 | -0.734 | -3.220 | 2.963 |  |
| Hs.2233 | NM_000759 | CSF3 | -3.951 | -3.023 | -0.876 | 1.266 |  |
| Hs.632586 | NM_001565 | CXCL10 | -3.827 | 0.713 | -2.901 | -0.095 |  |
| Hs.515146 | NM_016581 | ECSIT | 1.258 | -0.160 | 0.768 | -0.191 |  |
| Hs.131431 | NM_002759 | EIF2AK2 | -0.145 | -0.088 | 0.188 | 0.255 |  |
| Hs.181128 | NM_005229 | ELK1 | 1.339 | -0.270 | 0.678 | -0.406 |  |
| Hs.86131 | NM_003824 | FADD | -0.008 | -0.300 | -0.105 | -0.209 |  |
| Hs.728789 | NM_005252 | FOS | 1.210 | 0.512 | -1.390 | 0.018 |  |
| Hs.593339 | NM_002128 | HMGB1 | 0.763 | 0.094 | 1.178 | -0.148 |  |
| Hs.37003 | NM_005343 | HRAS | 0.864 | 0.144 | 0.972 | 0.034 |  |
| Hs.728810 | NM_005345 | HSPA1A | 0.080 | -0.319 | 0.575 | 0.779 |  |
| Hs.595053 | NM_002156 | HSPD1 | -0.092 | -0.073 | 0.556 | 0.146 |  |
| Hs.37026 | NM_024013 | IFNA1 | -1.703 | -0.272 | 0.198 | -0.418 |  |
| Hs.93177 | NM_002176 | IFNB1 | -3.770 | -0.690 | -2.111 | 2.029 |  |
| Hs.856 | NM_000619 | IFNG | NA | NA | NA | NA |  |
| Hs.597664 | NM_001556 | IKBKB | 0.323 | -0.389 | 0.147 | -0.557 |  |
| Hs.193717 | NM_000572 | IL10 | 0.303 | -0.022 | 1.985 | NA |  |
| Hs.673 | NM_000882 | IL12A | 0.041 | 0.494 | 0.416 | 0.660 |  |
| Hs.1722 | NM_000575 | IL1A | -1.555 | -0.200 | -0.801 | -0.280 |  |
| Hs.126256 | NM_000576 | IL1B | -2.871 | 0.224 | -0.225 | -1.060 |  |
| Hs.89679 | NM_000586 | IL2 | 0.173 | NA | NA | NA |  |
| Hs.654458 | NM_000600 | IL6 | -4.258 | -2.090 | -0.965 | 1.280 |  |
| Hs.624 | NM_000584 | IL8 | -0.396 | -0.106 | 0.676 | 0.030 |  |
| Hs.522819 | NM_001569 | IRAK1 | 1.954 | -0.019 | 1.636 | 0.051 |  |
| Hs.449207 | NM_001570 | IRAK2 | -0.533 | -0.954 | -0.724 | -0.119 |  |
| Hs.138499 | NM_016123 | IRAK4 | -0.117 | -0.198 | -0.365 | -0.232 |  |
| Hs.436061 | NM_002198 | IRF1 | -0.779 | -0.093 | -0.547 | 0.428 |  |
| Hs.75254 | NM_001571 | IRF3 | 0.126 | -0.144 | 0.793 | -0.318 |  |
| Hs.714791 | NM_002228 | JUN | 0.713 | 0.225 | 0.344 | 0.217 |  |
| Hs.36 | NM_000595 | LTA | -0.089 | 0.754 | -2.684 | -0.501 |  |
| Hs.653138 | NM_004271 | LY86 | 0.922 | NA | NA | NA |  |
| Hs.660766 | NM_015364 | LY96 | 0.962 | -0.103 | 1.773 | -0.305 |  |
| Hs.514012 | NM_002756 | MAP2K3 | 0.972 | 0.297 | 1.639 | -0.659 |  |
| Hs.514681 | NM_003010 | MAP2K4 | 0.543 | -0.392 | 0.473 | -0.033 |  |
| Hs.657756 | NM_005921 | MAP3K1 | 0.113 | 0.258 | 0.304 | -0.235 |  |
| Hs.644143 | NM_003188 | MAP3K7 | 0.194 | -0.144 | 0.400 | 0.032 |  |
| Hs.431550 | NM_004834 | MAP4K4 | -0.163 | -0.283 | 0.573 | 0.218 |  |
| Hs.138211 | NM_002750 | MAPK8 | 0.707 | -0.429 | 0.249 | -0.387 |  |
| Hs.207763 | NM_015133 | MAPK8IP3 | 0.901 | -0.268 | -0.330 | -0.088 |  |
| Hs.82116 | NM_002468 | MYD88 | -0.599 | 0.139 | 0.045 | 0.187 |  |
| Hs.654408 | NM_003998 | NFKB1 | -0.261 | -0.386 | -0.326 | -0.300 |  |
| Hs.73090 | NM_002502 | NFKB2 | -0.158 | -0.590 | -0.998 | -0.347 |  |
| Hs.81328 | NM_020529 | NFKBIA | -1.753 | -0.591 | -1.096 | 0.404 |  |
| Hs.2764 | NM_005007 | NFKBIL1 | 1.595 | 0.075 | 0.759 | -0.997 |  |
| Hs.530539 | NM_006165 | NFRKB | 2.273 | -0.065 | 0.317 | -0.236 |  |
| Hs.591667 | NM_003298 | NR2C2 | 0.412 | -0.424 | 0.229 | -0.312 |  |
| Hs.7886 | NM_020651 | PELI1 | 0.193 | 0.276 | 1.350 | -0.695 |  |
| Hs.103110 | NM_005036 | PPARA | 0.243 | 0.206 | 0.455 | -0.464 |  |
| Hs.570274 | NM_003690 | PRKRA | 0.359 | -0.243 | 0.391 | -0.025 |  |
| Hs.196384 | NM_000963 | PTGS2 | -0.139 | -0.640 | 0.393 | -0.364 |  |
| Hs.631886 | NM_002908 | REL | 0.822 | -0.508 | 0.040 | -0.618 |  |
| Hs.502875 | NM_021975 | RELA | -0.514 | -0.369 | -0.504 | 0.054 |  |
| Hs.103755 | NM_003821 | RIPK2 | -0.542 | -0.033 | 0.229 | 0.045 |  |
| Hs.532781 | NM_015077 | SARM1 | -0.391 | -0.168 | -0.152 | -0.372 |  |
| Hs.501624 | NM_021805 | SIGIRR | -0.461 | -0.713 | 0.173 | -0.442 |  |
| Hs.507681 | NM_006116 | TAB1 | 0.186 | -0.347 | 0.001 | -0.269 |  |
| Hs.505874 | NM_013254 | TBK1 | 1.957 | -0.179 | 2.030 | -0.666 |  |
| Hs.29344 | NM_182919 | TICAM1 | -0.955 | -0.118 | -0.707 | -0.038 |  |
| Hs.710895 | NM_021649 | TICAM2 | -0.156 | -0.258 | -0.503 | 0.162 |  |
| Hs.537126 | NM_001039661 | TIRAP | 0.610 | -0.250 | 0.312 | -0.551 |  |
| Hs.654532 | NM_003263 | TLR1 | -0.505 | 0.238 | -0.182 | -0.525 |  |
| Hs.120551 | NM_030956 | TLR10 | 2.619 | 2.334 | 4.054 | 1.813 |  |
| Hs.519033 | NM_003264 | TLR2 | -1.338 | -0.431 | -1.074 | 0.552 |  |
| Hs.657724 | NM_003265 | TLR3 | -1.284 | 0.145 | -0.674 | 0.239 |  |
| Hs.174312 | NM_138554 | TLR4 | -0.519 | -0.977 | 0.109 | 0.112 |  |
| Hs.604542 | NM_003268 | TLR5 | -0.782 | -0.831 | -1.771 | -0.940 |  |
| Hs.662185 | NM_006068 | TLR6 | 0.896 | -0.583 | 0.645 | -0.752 |  |
| Hs.659215 | NM_016562 | TLR7 | -0.794 | -1.039 | NA | -0.721 |  |
| Hs.660543 | NM_138636 | TLR8 | -0.014 | NA | NA | NA |  |
| Hs.87968 | NM_017442 | TLR9 | 1.133 | 0.343 | -0.947 | -0.728 |  |
| Hs.241570 | NM_000594 | TNF | -2.265 | 0.482 | -1.511 | 0.521 |  |
| Hs.279594 | NM_001065 | TNFRSF1A | 0.396 | -0.078 | 0.578 | -0.258 |  |
| Hs.368527 | NM_019009 | TOLLIP | 0.764 | -0.135 | 0.247 | -0.229 |  |
| Hs.591983 | NM_004620 | TRAF6 | 0.607 | -0.164 | 0.233 | -0.015 |  |
| Hs.524630 | NM_003348 | UBE2N | -0.058 | -0.121 | -0.086 | 0.190 |  |

**4.** TLR10 cellular localization upon overexpression

A549 cells were seeded into 8-well plates (Thermo Fisher Scientific, USA) at a density of 1 × 10⁴ cells per well. The cells were cultured in complete medium (DMEM with 10% FBS and 1% P/S). On the day of transfection, we transfected the cells as described in section 2.2-TLR10 overexpression. Two days later, the medium was removed, and the cells were washed three times with PBS and fixed with 4% paraformaldehyde for 10 minutes at room temperature. Following fixation, the samples were permeabilized for three minutes with 0.1% Triton-X100 and washed again three times for 10 minutes each with PBS. The cells were blocked in 0.5% BSA/PBS buffer for one hour, then incubated overnight at 4°C with primary antibodies (Ab1) in 0.5% BSA (see Table 7), under gentle shaking. The next day, the cells were washed three times with PBS and incubated for one hour with conjugated secondary antibodies (Ab2) in 0.5% BSA at room temperature. Afterward, the cells were washed again three times with PBS and incubated for three minutes in 4′,6-diamidino-2-phenylindole (DAPI) staining solution (DAPI:PBS = 1:10,000). Finally, the cells were washed three more times with PBS. The experiment was conducted in duplicate. Microscopic samples were analyzed using a Nikon Eclipse TE2000-U fluorescent microscope at 100x magnification.

Supplementary table 3: List of antibodies used in fluorescent microscopy.

| Target | Primary antibody (Ab1) | Secondary antibody (Ab2) |
| --- | --- | --- |
| TLR10 | Primary rabbit antibody against TLR10 (PRS3275, Sigma Aldrich) | Alexa-Fluor 488 (A32731, Thermo Fisher Scientific, ZDA |

Supplementary figure 2: Localization of TLR10 in A549 cells overexpressing *TLR10* (A549-TLR10_OE_) and A549 cells with native *TLR10* expression (A549 - control). After overexpression, TLR10 was mainly localised at the cell membrane, but to a lesser extent also in the cytoplasm (possibly in endosomes). Scale bar = 20 µm.
